# Predictivity of Autoimmune Stigmata for Gluten Sensitivity in Subjects with Microscopic Enteritis: A Retrospective Study

**DOI:** 10.3390/nu10122001

**Published:** 2018-12-18

**Authors:** Giuseppe Losurdo, Mariabeatrice Principi, Andrea Iannone, Antonio Giangaspero, Domenico Piscitelli, Enzo Ierardi, Alfredo Di Leo, Michele Barone

**Affiliations:** 1 Section of Gastroenterology, Department of Emergency and Organ Transplantation, University of Bari (Italy), Piazza G. Cesare 11, 70124 Bari, Italy; ianan@hotmail.it (A.I.); antoniogiangaspero@libero.it (A.G.); ierardi.enzo@gmail.com (E.I.); alfredo.dileo@uniba.it (A.D.L.); michele.barone@uniba.it (M.B.); 2 Section of Pathology, Department of Emergency and Organ Transplantation, University of Bari, Piazza G. Cesare 11, 70124 Bari, Italy; domenico.piscitelli@uniba.it

**Keywords:** gluten sensitivity, anti-gliadin, anti-nucleus antibody, autoimmunity, thyroiditis

## Abstract

Non-celiac gluten sensitivity (NCGS) is an emerging gluten-related condition. We investigated whether the presence of autoimmune stigmata in a group of patients with clinical suspicion of NCGS and a histological picture of microscopic enteritis (ME) could be a predictive factor of NCGS. Patients with ME were followed up by periodical examinations. At baseline, we collected data about previous clinical history, including autoimmune diseases. NCGS was diagnosed according to Salerno criteria; other causes of ME were diagnosed according to well-established protocols. Patients with celiac disease were excluded. Student’s and chi-square tests were used in univariate analysis. Kaplan-Meier curves and Cox regression were used to estimate hazard ratios (HR). Sixty-three patients were included. Twenty-two had a final diagnosis of NCGS; the remaining 41 had non-gluten-related causes of ME. Prevalence of autoimmune thyroiditis was higher among NCGS patients (40.1%) than in other ME (14.6%; *p* = 0.03). NCGS showed higher positivity rate for anti-gliadin (27.3% versus 2.5%; *p* = 0.006) and anti-nucleus (45.4% versus 12.2%; *p* = 0.005). Autoimmune thyroiditis had a non-significant trend (*p* = 0.06) for NCGS diagnosis, (HR = 2.4). Both anti-gliadin (HR = 2.4; *p* = 0.04) and anti-nucleus (HR = 2.7; *p* = 0.04) were directly associated with NCGS diagnosis. In conclusion, NCGS may have a cohort of autoimmune stigmata that can precede its diagnosis.

## 1. Introduction

Non-celiac gluten sensitivity (NCGS) is a gluten-related disorder in which ingestion of gliadin causes a rapid onset of gastrointestinal symptoms such as diarrhea, abdominal pain, discomfort or bloating [1]. The epidemiology of this disorder is uncertain, since the reported prevalence varies from 1 to 10% [2]. This wide range may be explained by the fact that self-diagnosis without an evaluation by a dedicated physician is very frequent. Therefore, such patients often start a gluten-free diet without medical advice. The clinical features of NCGS resemble that of irritable bowel syndrome and, therefore, several reports from literature have underlined that NCGS could be a form of irritable bowel syndrome induced by gluten ingestion [3]. Indeed, NCGS patients often show high somatization levels and are influenced by the “nocebo” effect [4]. For this reason, an expert consensus has developed a diagnostic strategy based on a double blind placebo controlled challenge with crossover [5]. This approach allowed scientists to distinguish self-reported gluten sensitivity from true NCGS, which accounts for the 14% of patients with NCGS self-diagnosis [6].

On the other hand, further evidence has demonstrated that NCGS has some peculiar immunologic characteristics. In about a half of cases, an increase in intraepithelial lymphocytes (IELs) in duodenal mucosa may be detected; therefore, NCGS can be classified under the “umbrella” term of microscopic enteritis [7,8,9,10,11]. Consequently, the enhanced infiltration of IELs witnesses a mucosal damage mediated by the immune system. The hypothesis of an immune-mediated mechanism for the pathogenesis of NCGS has been confirmed by further evidence. Indeed, it has been demonstrated that the involvement of the innate immune system could play a central role in NCGS, while acquired immunity has a negligible importance. The research has focused therefore on the evaluation of possible markers of native immunity in NCGS. In two relevant papers, Sapone et al. [12,13] confirmed this hypothesis by showing an increase in mRNA codifying toll like receptors (TLR)-2. In detail, TLRs are receptors activated by nonself-antigens during innate immune response. More recently, it has been shown that CD14 and lipopolisaccharide binding protein levels were higher in blood samples of patients with NCGS compared to healthy controls and celiac disease [14]. These molecules are indicators of innate immune activation against bacterial antigens, thus confirming the original pathogenetic pathway of this disease.

On these bases, an autoimmune starting point could be considered in NCGS similarly to celiac disease. Therefore it is hypothesizable that NCGS could be associated with autoimmune disorders. However, this issue has been poorly investigated so far. Indeed, a pivotal study performed in a cohort of 131 NCGS patients [15] showed a prevalence of autoimmune diseases of 29%, significantly higher than that in the control group (4%, *p* < 0.001). Additionally, this study demonstrated that anti-nucleus antibody (ANA) positivity, a well-known marker of autoimmune setting, was present in 46% of NCGS subjects, compared to 2% of controls, and ANA positivity correlated with DQ2/8 haplotypes. Furthermore, Hashimoto thyroiditis was the most frequently reported autoimmune disease, being found in 29 out of the 40 patients with coexisting autoimmune phenomena. However, to the best of our knowledge, no study has investigated the possibility that association with autoimmune disorders could forego the onset of NCGS. Therefore, the aim of this study was to elucidate whether the presence of autoimmune stigmata in a group of patients with clinical suspicion of NCGS and a histological picture of microscopic enteritis could be a factor predicting the future development of NCGS.

## 2. Methods

### 2.1. Patients Selection

We retrospectively enrolled patients with a clinical suspicion of NCGS and a duodenal picture of microscopic enteritis attending our outpatient unit in the period from January 2013–2017.

All patients at baseline underwent upper endoscopy and duodenal biopsy sampling that showed a picture of microscopic enteritis according to the Bucharest consensus [16]. At baseline, we collected data about previous clinical history, including existence of autoimmune diseases and drug consumption. In all cases, autoimmunity assay for ANA, IgG anti-gliadin antibodies (AGA), anti-transglutaminase, anti-endomysium, Anti-Smooth Muscle Antibodies, liver kidney microsoma antibodies, Anti-mitochondrial antibodies was performed. Titers > 1:80 were considered significant. Finally, genotyping for human leukocyte antigen (HLA) DQ2 and DQ8 haplotypes was carried out.

Subjects with a diagnosis of celiac disease or wheat allergy or with positivity to anti-transglutaminase or anti-endomysium antibodies were excluded because the aim of the study was to find predictive factors of NCGS in comparison to a group of patients with microscopic enteritis with or without gluten-related conditions. Therefore, the group of patients with non-gluten-related causes of microscopic enteritis was the control group.

### 2.2. Histology and Immunohistochemistry

For each patient, formalin embedded biopsy samples of distal duodenum, performed at baseline, were retrieved. Histological examination was carried out on Hematoxylin-Eosin stained sections. Immunohistochemistry of CD3 lymphocytes was performed using monoclonal murine antibody (Novocastra Leica Biosystems Ltd., Newcastle, UK), according to the manufacturer’s instructions. In all subjects, IELs were counted in a field containing at least 1000 enterocytes and were expressed as number per 100 enterocytes. The count was carried out in the epithelial layer by two observers in a blinded fashion. Collection and processing were managed according to BRISQ recommendations [17].

### 2.3. Follow Up

The follow-up approach aiming to establish a final diagnosis has been previously described [18]. In particular, according to medical judgment, patients underwent a series of laboratory testing when required (repetition of serology for celiac disease, full blood count, folate, vitamin B12, serum protein electrophoresis with immunoglobulin subclasses, stool investigations, fecal occult blood test, fecal calprotectin, urea/lactose/glucose breath test). When necessary, colonoscopy with random biopsy samples or a second upper endoscopy was performed and, eventually when required, a magnetic resonance enterography. All patients gave informed consent to undergo invasive procedures and to give, in anonymous form, their clinical data for research purposes.

Celiac disease was diagnosed if duodenal biopsy showed a microscopic picture of Marsh 1 or higher, along with the positivity of IgA anti tissue transglutaminase 2 antibodies, according to current guidelines [19]. Wheat allergy was tested by the skin-prick test and Radio Allergo Sorbent Test (RAST). As reported in “patient selection” criteria, when a subject was diagnosed with celiac disease or wheat allergy during the follow up period, he/she was excluded from the final analysis, according to the protocol of the study.

The diagnosis of NCGS was made according to the Salerno criteria [5]. Irritable bowel syndrome was diagnosed if the Rome III criteria were fulfilled when all investigations excluded other organic diseases [20].

During the follow up period, all patients were under a gluten containing diet, until the final diagnosis was established.

### 2.4. Statistical Analysis

Comparisons among continuous data were performed by Student’s *t* test, after having assessed normality by the Kolmogorov-Smirnov test, while categorical variables were analyzed by Fisher’s exact test.

Kaplan-Meier curves were plotted to estimate median diagnostic time for two groups (diagnosis of NCGS according to presence/absence of autoimmunity signs) and the log-rank test was applied. For multivariate analysis, the Cox regression model was used. Estimates of risk were expressed as Hazard Ratios (HR) and the 95% confidence intervals (CI) of HR were calculated. All statistical tests were 2-tailed and were performed at the 5% level of significance. The statistical analysis was performed using the software SPSS Statistics for Windows, Version 21.0. Armonk, NY, USA: IBM Corp.

## 3. Results

### 3.1. Patient Characteristics

Eighty-six patients with a histological picture of microscopic enteritis were initially selected. Of these, 23 developed celiac disease during the follow up and were excluded according to our protocol. No case of wheat allergy was recorded. Therefore, 63 patients were included in our final analysis. These patients were followed up for a median time of 18 months (range 12–64 months). Their mean age was 34.1 ± 12.1 years. Fifty of them (79.4%) were female. Thirty-two patients (50.8%) had a grade 1 enteropathy according to Marsh staging.

Nineteen patients had an associated autoimmune disease. The most common disorder was autoimmune thyroiditis (15 subjects, all with Hashimoto thyroiditis; no case of Graves disease was found), while single cases of scleroderma, psoriasis, Crohn’s disease and lymphocytic colitis were respectively recorded. Seven patients were AGA positive, while 15 cases of ANA positivity were found (titer 1:80 in all cases). We did not find positivity for any other tested autoantibodies.

**Figure 1:**
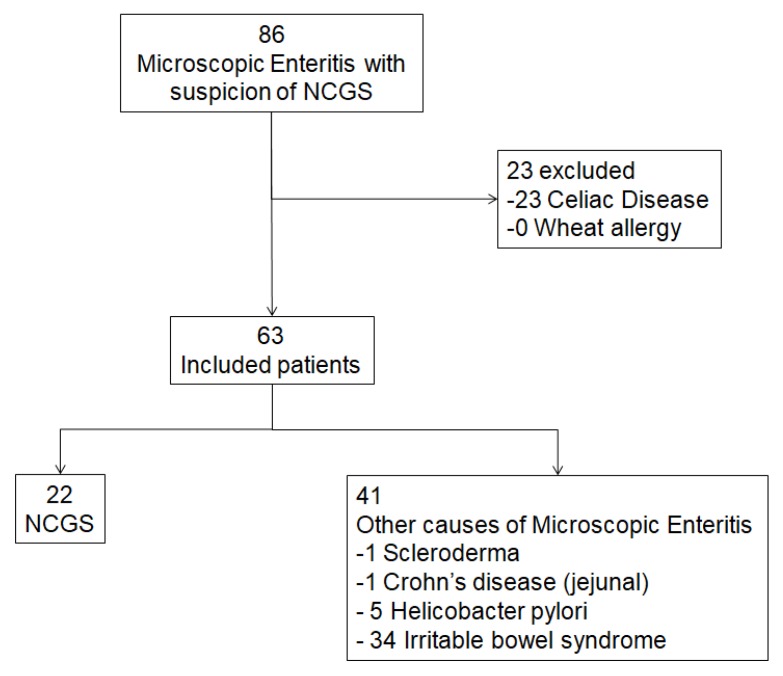
Flow chart showing the process of patient selection according to inclusion and exclusion criteria. NCGS: non-celiac gluten sensitivity.

At the end of the follow-up time, 22 patients were diagnosed of NCGS according to the Salerno criteria, while the remaining 41 had non gluten-related causes of microscopic enteritis: 34 irritable bowel syndrome, 5 *Helicobacter pylori* (*H. pylori*) infection, one scleroderma and one jejunal Crohn’s disease. A flow chart summarizing the process of patient selection is reported in Figure 1.

In the comparison between NCGS and other patients without a gluten-related disorder (Table 1), the prevalence of autoimmune thyroiditis was higher among NCGS patients (40.1%) than in other patients (14.6%, *p* = 0.03). Furthermore, we found in NCGS a higher baseline positivity rate for AGA (27.3% versus 2.5%, *p* = 0.006) and ANA autoantibodies (45.4% versus 12.2%, *p* = 0.005). NCGS showed a Marsh 1 grade in the 68.2%, versus the 41.5% in the other group, with a *p* = 0.07, close to significance. Subjects with NCGS carried more frequent HLA DQ2 and DQ8 haplotypes (95.4% versus 58.5%, *p* = 0.002). Finally, from a symptom point of view, in NCGS, weight loss or headache was found more frequently than in other non gluten-related microscopic enteritis.

### 3.2. Predictive Factors for NCGS Onset

**Figure 2:**
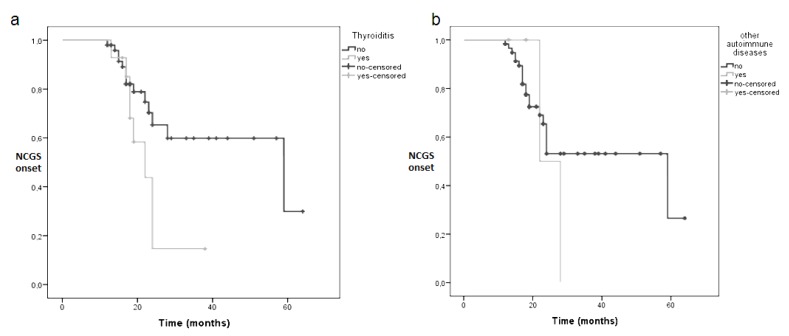
Kaplan-Meier curves showing the influence of autoimmune thyroiditis (**a**) or other autoimmune disorders (**b**) as factors predictive of NCGS development. *p* log rank = 0.045 for autoimmune thyroiditis and *p* log rank = 0.54 for other autoimmune diseases.

Patients with autoimmune thyroiditis encountered an earlier diagnosis of NCGS (median 27 months, range 15–28 months) than those without thyroiditis (median 59 months, range 14–103 months), with a statistical difference, demonstrated by a *p* log rank = 0.045, as reported in Kaplan-Meier curve in Figure 2a. The presence of autoimmune diseases other than autoimmune thyroiditis did not influence the timing of NCGS diagnosis. Median diagnostic time was 22 months if an autoimmune disease other than thyroiditis was present and 59 months if absent, *p* log rank = 0.54 (see Figure 2b).

**Figure 3:**
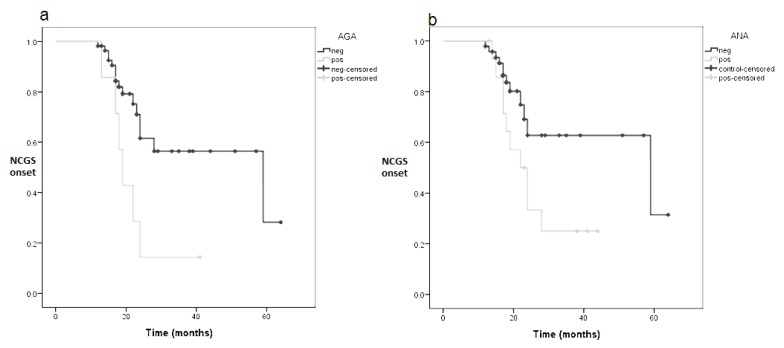
Kaplan-Meier curves showing the influence of AGA (**a**) or ANA (**b**) as factors predictive of NCGS development. *p* log rank = 0.012 for AGA and *p* log rank = 0.036 for ANA. AGA: anti-gliadin antibodies; ANA: anti-nucleus antibodies.

AGA positivity led to an early NCGS diagnosis (median 19 months, range 16–21versus 59 months, range 14–103, *p* log rank = 0.012). The Kaplan-Meier curve of this analysis is reported in Figure 3a. Similarly, ANA presence was a predictive factor for early NCGS diagnosis: the median diagnostic time was 22 months (range 16–27) for ANA positivity and 59 months (range 9–108) for ANA negativity, *p* log rank = 0.036, as shown in Figure 3b.

Multivariate Cox analysis showed that autoimmune thyroiditis had a non-significant trend (*p* = 0.06) demonstrating an increased risk for NCGS diagnosis, with an HR = 2.4 (95% CI 0.9–5.8). On the other hand, both ANA (HR = 2.4, 95% CI 1.1–5.7, *p* = 0.04) and AGA (HR = 2.7, 95% CI 1.1–7.1, *p* = 0.04) were directly associated to NCGS diagnosis. Among the other variables that showed significance at univariate analysis in Table 1, only headache showed an association with NCGS (HR = 4.5, 95% CI 1.7–11.8, *p* = 0.002), while HLA status and weight loss were not significant factors. Further details of multivariate analysis are reported in Table 2.

### 3.3. Autoimmunity and HLA Status

**Figure 4:**
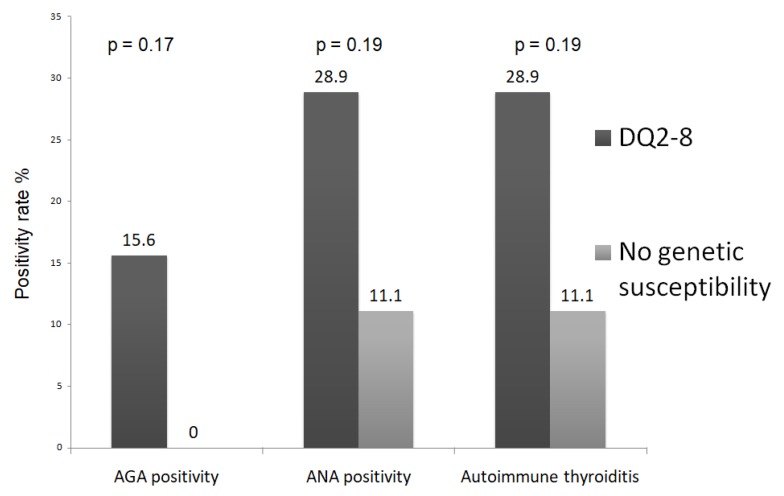
Prevalence of AGA, ANA and autoimmune thyroiditis in patients with or without DQ2-8 haplotypes. The analysis refers to the whole sample size of 63 patients.

Forty-five out of 63 (71.4%) enrolled patients carried a DQ2-8 haplotype. In DQ2-8 positive subjects, we found a higher AGA positivity (15.6% versus 0%, *p* = 0.17) when compared with DQ2-8 negative patients, as well as higher rates of ANA positivity (28.9% versus 11.1%, *p* = 0.19) and autoimmune thyroiditis (28.9% versus 11.1%, *p* = 0.19), despite that in all cases proportions did not reach significance (see Figure 4). If we consider only the 22 NCGS patients, 21 of them had HLA DQ2-8. The positivity rate in such patients of AGA, ANA and thyroiditis was 28.6%, 42.8% and 38.1% respectively. The only patients without DQ2-8 were ANA positive and had autoimmune thyroiditis; however, we did not perform any statistical test because the sample size of the two groups was uneven (21 versus 1 patient).

## 4. Discussion

The diagnosis of NCGS is often difficult, since it is based on clinical features and requires the exclusion of other diseases that show a similar picture, such as mild celiac disease, seronegative or potential celiac disease, immunoglobulin deficiencies or food allergy [21,22,23,24,25,26,27]. Moreover, a reliable diagnostic marker it far from being found.

On these bases, the search for indirect signs of NCGS would be very useful to improve the diagnosis. Autoimmune disease clustering is very common in celiac disease; therefore, it is presumable that even NCGS could be associated with other autoimmune disorders [28]. Indeed, there are several reports in literature demonstrating connection with psoriasis, dermatitis herpetiformis [29,30], axial spondyloarthritis and connective tissue diseases [31].

To the best of our knowledge, only one study investigated the possibility of autoimmune involvement in NCGS [15]. In detail, a higher prevalence of autoimmune diseases (in particular autoimmune thyroidits) and ANA positivity were found in comparison to the control group of irritable bowel syndrome patients. This finding is substantially in agreement with our results. However, some aspects should be underlined. The previous study was a transversal cohort analysis aimed to investigate the prevalence of autoimmune phenomena in NCGS, while the present study was a follow up investigation with the aim of seeking predictive factors of NCGS. Therefore, it provides a “dynamic scenario” of microscopic enteritis. Moreover, the study by Carroccio included a positive control group of celiac patients. We decided to exclude celiac disease since the diagnosis of NCGS is often challenging in comparison to non-gluten related microscopic enteritis, such as irritable bowel syndrome and even *H. pylori* infection [32,33]. On the other hand, the diagnosis of celiac disease is based on villous atrophy and serological specific markers [34] and is known to be frequently associated with autoimmune diseases. Therefore, since the differential diagnosis of non-gluten-related causes of microscopic enteritis is much easier versus celiac disease than versus NCGS, the inclusion of patients with celiac disease in our analysis would have not been relevant for the purpose of the study.

Autoimmune thyroiditis was very common in our population of NCGS. An Italian study showed a prevalence of 24% for celiac disease, and it was the most common autoimmune disorder associated with celiac disease with an odds ratio of 2.55 [35]. Some authors claim that, since the thyroid gland shares with the gut a common embryonic origin during fetal development, a cross reaction for auto-antibodies may occur [36]. Moreover, some genetic polymorphisms are common in both diseases (i.e., FOXP3, CD25, CD40, CTLA-4, the HLA genes, PTPN22) [37]. Finally, an alteration of a thyroid-gut axis due to microbiota perturbation has been hypothesized [38,39]. Despite that we demonstrated that autoimmune thyroiditis is very common in NCGS, the multivariate analysis did not confirm that it could forego NCGS development; however, the level of significance (*p* = 0.06) illustrates a suitable trend, so the small sample size could explain this result. ANAs are not very frequent in celiac disease, and they often have an uncertain meaning due to low titers; however, they gain a certain significance when other autoimmune disorders coexist [40]. Therefore we believe that it could be a further clue of the autoimmune origin of NCGS, with a certain predictive value. Finally, the role of AGA in NCGS has been already widely discussed, since AGA are present in about a half of cases [41] in complete agreement with our finding; moreover, we showed that they may even have a prognostic value.

Some issues seem to be in contrast with other evidence from literature. For example, a Marsh 1 stage is very common in NCGS and it has been already demonstrated to be a predictive factor of NCGS among duodenal lymphocytosis [18]. Although in our report we found a higher presence of Marsh 1 grade in NCGS than in other microscopic enteritis (68.2% versus 41.5%), we did not find statistical significance (*p* = 0.07), possibly due to the small sample. However, this result supports enhanced inflammatory response with an autoimmune mark in NCGS [42].

The role of HLA in NCGS deserves further discussion. In a previous study [15], patients carrying the DQ2-8 haplotype showed a non-significant trend of having autoimmune thyroiditis and ANA. Herein, we also showed that this haplotype was more common if patients had AGA, ANA or autoimmune thyroiditis, but statistical significance was not reached, as shown in Figure 4. Moreover, the DQ2-8 was more common in NCGS in the univariate analysis of Table 1, but lacked significance in multivariate analysis. Again, the small sample size could be a limitation. However, in this perspective, considerable evidence showed that the DQ2-8 haplotype could be associated with autoimmune disorders other than celiac disease, such as type 1 diabetes [43] and autoimmune thyroiditis [44].

Finally, headache was the clinical symptom which showed the strongest predictive value for NCGS (HR = 4.5). Indeed, it has been frequently described as an extraintestinal manifestation, with a prevalence of 54% and a rapid improvement after a gluten-free diet [45,46]. Some studies proposed that the inflammatory reaction in celiac disease, with the up-regulation of certain cytokines, may play a role [47]. In addition, both molecular mimicry and intermolecular help (a process by which immune reactivity to one molecule can trigger an immune response to another) have been proposed as potential mechanisms by which gluten ingestion can result in damage to the central nervous system [48].

The present study has some limitations due to the retrospective nature of the analysis. Indeed, the low number of NCGS patients is the most important one. Additionally, the diagnosis of autoimmune thyroiditis was not performed in our center; therefore, it was based on the clinical history of the patient. A such, we could not collect sufficient data about thyroid hormones levels and thyroid autoimmunity at the moment of patient enrollment.

## 5. Conclusions

In conclusion, our study demonstrated that NCGS may have a cohort of autoimmune stigmata that can precede its diagnosis and may have a predictive value. This could be helpful, since a biological marker for NCGS has not been discovered so far [49], and autoimmunity investigations could support the diagnosis in some cases. If we consider that the most common causes of microscopic enteritis are irritable bowel syndrome, *Helicobacter pylori* infection, drugs and bacterial/viral infections, the presence of such autoimmune stigmata is anything but representative of these conditions. Therefore, it may only help differential diagnosis. However, when microscopic enteritis underlies autoimmune connective tissue disorders as well as vasculitides, it is presumable that our results could not be helpful for differential diagnosis. Our results suggest that ANA and AGA positivity, headache and, with a slighter evidence, autoimmune thyroiditis, are positively associated with a diagnosis of NCGS in a setting of patients with microscopic enteritis. However, at the moment, our results show only a possible association and cannot demonstrate that they are authentic predictors of NCGS development. Indeed, the present study is a single center experience based on a small number of patients which needs to be confirmed in large trials. Therefore, we can assert that it would be appropriate to suspect NCGS and propose a diagnostic protocol according to Salerno criteria in patients with microscopic enteritis showing the above reported stigmata of autoimmunity. In conclusion, due to the limitations of our study (small sample size and retrospective analysis), other prospective studies are warranted to confirm whether ANA, AGA, headache and autoimmune thyroiditis are predictors of NCGS onset and ascertain whether a gluten-free diet could be beneficial for both NCGS symptoms and extra-intestinal autoimmune conditions. In this regard, it is interesting to highlight a recent study demonstrating that a gluten-free diet may bring clinical benefits to women with autoimmune thyroid disease by reducing anti-thyroid antibody titers [50].

However, we are conscious that further studies conducted on large sample sizes and with a prospective design are warranted.

## Figures and Tables

**Table 1:** Comparison of demographic and clinical features of NCGS versus non-gluten-related microscopic enteritis patients (univariate analysis).

	Non Gluten-Related Microscopic Enteritis (*n* = 41)	NCGS (*n* = 22)	*p* Value
**Age (mean ± standard deviation)**	36.0 ± 13.3	30.6 ± 8.7	0.09
**Female sex, *n* (%)**	31 (75.6)	19 (86.4)	0.51
**Marsh classification** **Marsh 0** **Marsh 1**	24 (58.5) 17 (41.5)	7 (31.8) 15 (68.2)	0.07
**AGA, *n* (%)**	1 (2.5)	6 (27.3)	0.006
**ANA, *n* (%)**	5 (12.2)	10 (45.4)	0.005
**Familiarity for celiac disease, *n* (%)**	0 (0)	2 (9.1)	0.12
**Autoimmune thyroiditis, *n* (%)**	6 (14.6)	9 (40.1)	0.03
**Other autoimmune diseases, *n* (%)**	2 (4.9)	2 (9.1)	0.61
**Anemia, *n* (%)**	7 (17.1)	3 (13.6)	0.70
**Folate deficit, *n* (%)**	6 (14.6)	7 (31.8)	0.19
**HLA, *n* (%)** **DQ2-8** **No susceptibility**	24 (58.5) 17 (41.5)	21 (95.4) 1 (4.6)	0.002
**Weight loss, *n* (%)**	8 (19.5)	10 (45.4)	0.04
**Bloating, *n* (%)**	22 (53.6)	17 (77.3)	0.10
**Diarrhea, *n* (%)**	7 (17.1)	9 (40.1)	0.08
**Tiredness, *n* (%)**	7 (17.1)	8 (36.4)	0.12
**Headache, *n* (%)**	0 (0)	11 (50)	<0.001

AGA: anti-gliadin antibodies; ANA: anti-nucleus antibodies; HLA: human leukocyte antigen.

**Table 2:** Multivariate Cox model analyzing factors associated with NCGS development.

	HR (95% CI)	*p* Value
**Headache**	4.5 (1.7–11.8)	0.002
**Weight loss**	1.7 (0.4–7.2)	0.45
**HLA DQ2-8**	6.6 (0.8–53.5)	0.07
**AGA**	2.7 (1.1–7.1)	0.04
**ANA**	2.4 (1.1–5.7)	0.04
**Autoimmune thyroiditis**	2.4 (0.9–5.8)	0.06

AGA: anti-gliadin antibodies; ANA: anti-nucleus antibodies; CI: confidence interval; HLA: human leukocyte antigen; HR: hazard ratio.
